# Temporal brain dynamics and fluid reasoning after extreme prematurity

**DOI:** 10.1093/cercor/bhag174

**Published:** 2026-10-19

**Authors:** Nelly Padilla, Anira Escrichs, Elvira del Agua, Morten Kringelbach, Antonio Donaire, Gustavo Deco, Ulrika Åden

**Affiliations:** Department of Women’s and Children’s Health, Karolinska Institutet, Tomtebodavägen 18A, Stockholm S-171 77, Sweden; Computational Neuroscience Group, Center for Brain and Cognition, Department of Information and Communication Technologies, Universitat Pompeu Fabra, Roc Boronat 138, 08018, Barcelona, Spain; Computational Neuroscience Group, Center for Brain and Cognition, Department of Information and Communication Technologies, Universitat Pompeu Fabra, Roc Boronat 138, 08018, Barcelona, Spain; Department of Psychiatry, University of Oxford, Warneford Hospital, Warneford Ln, Oxford OX3 7JX, Storbritannien, United Kingdom; Center for Music in the Brain, Aarhus University Hospital Nørrebrogade 44, Building 10G, 4th and 5th floor, 8000 Aarhus C, Denmark; Department of Neurology, Institute of Neuroscience, Hospital Clinic, Universidad de Barcelona and Institut D’investigacions Biomèdiques August Pi i Sunyer (IDIBAPS), Villarroel 170, 08036 Barcelona, Spain; Biomedical Research Networking Center in Bioengineering, Biomaterials and Nanomedicine (CIBER-BBN), Instituto de Salud Carlos III, Av. Monforte de Lemos 3–5, Pabellón 11, Planta 0, 28029 Madrid, Spain; Computational Neuroscience Group, Center for Brain and Cognition, Department of Information and Communication Technologies, Universitat Pompeu Fabra, Roc Boronat 138, 08018, Barcelona, Spain; School of Psychological Sciences, Monash University, Melbourne, Clayton, VIC 3800, Australia; Institució Catalana de la Recerca i Estudis Avancats (ICREA), Passeig Lluís Companys 23, 08010, Barcelona, Catalonia, Spain; Department of Neuropsychology, Max Planck Institute for human Cognitive and Brain Sciences, Stephanstraße 1A, 04103 Leipzig, Germany; Department of Women’s and Children’s Health, Karolinska Institutet, Tomtebodavägen 18A, Stockholm S-171 77, Sweden; Department of Neonatology, Karolinska University Hospital, Eugeniavägen 23, SE-171 64, Stockholm, Sweden; Department of Biomedical and Clinical Sciences, Linköping University, Campus US, 581 83, Linköping, Sweden

**Keywords:** brain dynamics, cognitive development, extreme prematurity, fluid reasoning, temporal irreversibility

## Abstract

Extremely preterm (EPT) birth (<28 weeks’ gestation) disrupts brain maturation and increases later neurodevelopmental and mental health vulnerability. Temporal irreversibility quantifies directional brain dynamics, capturing the temporal organization of brain function. We examined whether EPT birth alters temporal irreversibility at 10 years and its association with cognition at 12 years. Resting-state functional MRI was acquired at 10 years of age in 33 children born EPT and 26 term-born controls. At 12 years of age, 29 children born EPT and 21 term-born controls underwent cognitive assessment using the Wechsler Intelligence Scale for Children-Fifth edition (WISC-V). Temporal irreversibility was quantified globally, across large-scale functional networks (visual, somatomotor, dorsal attention, salience, limbic, control, and default mode [DMN]), and at the node level using the INSIDEOUT framework. Children born EPT showed reduced temporal irreversibility globally and in higher-order association networks (control and DMN, *P* < 0.01; dorsal attention and salience, *P* < 0.05). Node-level analyses identified two association-network parcels that remained significant after false discovery rate correction (left precuneus and right dorsomedial frontal cortices). Within the EPT group, fluid reasoning was the only WISC-V domain significantly associated with temporal irreversibility, with higher global irreversibility associated with poorer fluid reasoning. These findings may inform future neurodevelopmental monitoring and intervention strategies after prematurity.

## Introduction

The neonatal period represents a critical window for human brain development, during which large-scale neural circuits that support motor, cognitive, socio-emotional, and language functions are established (Cara et al. 2025; Li et al. 2026). Infants born extremely preterm (EPT, <28 weeks of gestation) are exposed to the extrauterine environment at a developmental stage when key neurodevelopmental processes (including dendritic differentiation, synaptogenesis, myelination, axonal growth, and axonal pruning) are still unfolding (Kostović et al. 2019). Disruption of these processes increases risk for later cognitive and behavioral impairments (Serenius et al. 2023; Kvanta et al. 2024), with accumulating evidence also indicating elevated risk for mental health problems (Twilhaar and Wolke 2025). Studies consistently demonstrate that children born very preterm show substantial average deficits in cognition and academic outcomes compared with term-born peers (Serenius et al. 2013; Pascoe et al. 2021; Behboodi et al. 2025), underscoring the need for biomarkers that improve our understanding of neurodevelopmental and psychiatric vulnerability.

Functional brain networks emerge across overlapping prenatal and postnatal periods and mature in a hierarchical pattern: primary sensory and motor systems consolidate early, followed by socio-emotional networks and, later, higher-order association systems (Gao 2025). Critically, this hierarchy is not only spatial but also temporal. Information flows directionally across timescales, progressing from fast, local dynamics in sensory cortices toward slower, integrative processes in higher-order association networks (Deco et al. 2022; Kringelbach et al. 2024). This temporal directionality, often referred as the brain's “arrow of time,” is a hallmark of hierarchical organization and efficient information processing. If extreme prematurity disrupts the normal maturation of this hierarchy, alterations would be expected not only in connectivity strength but also in the temporal organization of neural activity.

Temporal irreversibility provides a principled metric for quantifying neural directionality by capturing the extent to which brain activity unfolds in a time-asymmetric manner, reflecting nonequilibrium dynamics and hierarchical temporal organization. Formally, irreversibility quantifies how distinguishable a neural-time series is from its time-reversed counterpart: high irreversibility reflects directional, nonequilibrium dynamics, at the system level, whereas low irreversibility indicates more symmetric, reversible fluctuations (Deco et al. 2022; Kringelbach et al. 2023). Recent work applying time-reversal asymmetry measures to neural recordings links higher irreversibility to richer hierarchical processing, whereas reduced irreversibility has been observed in neurological disorders (Cruzat et al. 2023; Sadeghi et al. 2025), and disorders of consciousness (Guzmán et al. 2023), consistent with diminished dynamical complexity. Temporal irreversibility therefore complements standard connectivity and variability measures by explicitly indexing the temporal arrow of brain activity, rather than a specific anatomical or physiological mechanism. It captures a property distinct from the propagation and integration measured by intrinsic ignition and the variability in large-scale coordination measured by metastability.

Prior imaging studies, including children born EPT, report weaker signal propagation and reduced metastability, particularly within higher-order association networks such as the default mode, salience, and frontoparietal networks (Padilla et al. 2020; Padilla et al. 2023), but have not tested whether these alterations reflect a disruption of temporal directionality. Temporal irreversibility provides a complementary approach to test whether these previously described alterations in large-scale brain dynamics are accompanied by changes in their time-asymmetric temporal organization.

In this study, we examined temporal irreversibility in 10-year-old children from a longitudinal cohort of EPT and term-born controls, extending previous work in this cohort by investigating the time-asymmetric organization of large-scale brain dynamics. Our aims were: (1) to quantify temporal irreversibility globally and within canonical resting-state networks (salience, dorsal attention, control, default mode, limbic, visual, and somatomotor) using time-reversal asymmetry of blood oxygenation level-dependent (BOLD) signals; (2) to test whether children born EPT exhibit reduced irreversibility, with the strongest effects in higher-order association networks; and (3) to examine whether temporal irreversibility is associated with poorer cognitive performance. We hypothesized that children born EPT would exhibit reduced temporal irreversibility, particularly in higher-order association networks, and examined the relationship between irreversibility and cognitive performance without prespecifying the direction of this association.

## Materials and methods

Participants were recruited from the Swedish population-based EXPRESS cohort, a prospective study of children born EPT in Sweden (EXPRESS Group et al. 2009). The regional cohort included 112 children born EPT at less than 27 weeks’ gestation in Stockholm County, between 2004 and 2007. Infants were scanned at term-equivalent age. At approximately 10 years of age, children were invited for a follow-up magnetic resonance imaging (MRI) scan, which constituted the primary assessment of the present study. As part of the original longitudinal study design, participants also underwent a developmental assessment at 12 years of age.

A total of 66 children born EPT and 46 term-born controls underwent the 10-year MRI assessment (Supplementary Fig. S1). The characteristics of the cohort have been described previously (Padilla et al. 2020). In brief, infants excluded from this study were those with congenital infections and malformations. Furthermore, only infants without severe brain lesions (ie, intraventricular hemorrhage grades I–II and mild white matter abnormalities, qualitatively defined by MRI examinations at term-equivalent age) (Skiöld et al. 2010), were included. The exclusion criteria for the term-born controls were identical, with the additional exclusion of any history of birth complications. After exclusion of participants with brain abnormalities (7 EPT and 2 term-born controls) and low-quality resting-state fMRI data, including excessive motion and other image quality issues (26 EPT and 18 term-born controls), the final study sample comprised 33 children born EPT and 26 term-born controls (Supplementary Fig. S1).

A priori sample size calculations were not performed. The sample size was determined by the number of children available from the population-based EXPRESS cohort who were born in Stockholm, who survived to school age and had high-quality resting-state functional MRI data suitable for analysis. No additional eligible participants were available. The regional ethical review board in Stockholm granted ethical approval for the study (04–889/2, 2010/850–31/1, 2014/1035–32, and 2016/17), and written, informed consent was obtained from the parents of the children. The neonatal characteristics of the two groups are presented in Supplementary Table S1.

### Cognitive assessment

Children underwent a cognitive assessment at 12 years of age using the Wechsler Intelligence Scale for Children-Fifth edition (WISC-V). The assessment yields five indices (fluid reasoning, working memory, processing speed, verbal comprehension index, and visual spatial ability) which together provide the full-scale intelligence quotient.

### MRI data acquisition

The conventional MRI protocol has previously been described (Padilla et al. 2020). During scanning, children were instructed to keep their eyes closed, remain as motionless as possible, and stay awake throughout the resting-state acquisition. MRI examinations were performed without sedation. The MRI technician maintained communication with the participants between imaging sequences and continuously supervised the examination. Although no objective measures of vigilance (eg, eye tracking or electroencephalography (EEG)) were acquired, no participant was observed to fall asleep during scanning. Briefly, MRI data were acquired using a Sigma HDx 3 T MR scanner (GE Healthcare). The MRI protocol included a sagittal 3D-T1 weighted with a BRAVO SPGR sequence: time to inversion = 400 ms, field of vision = 240 × 240 mm^2^, flip angle = 12°; voxel size 1 × 0.938 × 0.938 mm^3^; slice thickness = 1.0 mm. Resting state functional data (rs-fMRI) were acquired, namely: GE-EPI, repetition time (TR)/time echo = 2000/30 ms; flip angle = 70°; voxel size 3.0 × 3.0 × 3.5 mm^3^ with full-brain coverage; 300 volumes.

### Functional MRI preprocessing and motion censoring

The rs-fMRI dataset was pre-processed using FSL software, version 5.0.5 (FMRIB). All the raw rs-fMRI data and outputs from each preprocessing step were examined visually. Participants with poor-quality rs-fMRI data due to excessive motion were excluded before further analyses. In addition to the initial steps (slice-timing correction, volume realignment, geometric distortion correction using reversed encoding images, co-registration of the functional image to the T1-weighted image, and registration from high-resolution structural data to the study-specific template), framewise displacement (FD; threshold of 0.2 mm), and the temporal derivative of time courses (DVARS) were calculated as quality-control measures. Runs with more than 15% outlier volumes according to FD or DVARS were excluded. Importantly, volumes flagged by FD and DVARS were not removed from retained runs. No volume scrubbing, censoring, interpolation, or concatenation were performed, thereby preserving the continuous temporal structure required for the temporal irreversibility analyses. Residual motion-related components were identified using Independent Component Analysis–based Automatic Removal Of Motion Artifacts (ICA-AROMA) and removed by regression from the continuous time series. Mean white-matter and cerebrospinal-fluid signals were also calculated from eroded tissue masks, as previously described. Although these preprocessing steps reduce major nonneural confounds, residual physiological, vascular, scanner-related, and other nonneural contributions to the BOLD signal cannot be completely excluded. Lastly, a band-pass temporal filter with a cutoff frequency of 0.01–0.07 Hz was applied, consistent with our previous studies of brain dynamics in children born EPT (Padilla et al. 2020; Padilla et al. 2023), to retain slow spontaneous BOLD fluctuations while minimizing higher-frequency physiological noise.

We used the extracted and averaged time courses of seven neural networks defined using the Schaefer parcellation atlas (Schaefer et al. 2018): the salience, dorsal attention, control, default mode, limbic, visual, and somatomotor networks (Supplementary Table S1).

### Quantification of temporal irreversibility using the INSIDEOUT framework

An overview of the framework is shown in Fig. 1. Figure 1a illustrates the conceptual distinction between forward and reversed temporal evolution, and Fig. 1b shows its implementation using lagged brain time series. In this framework, temporal irreversibility is quantified as the asymmetry between pairwise temporal dependencies in the forward and time-reversed signals, computed as the difference between the time-shifted correlation of the original time series and those of their artificially reversed versions (Deco et al. 2022) (Fig. 1a and b). The rationale is that temporal dependencies between BOLD time series vary as a function of the time shift (*τ)*. If these temporal dependencies are directionally asymmetric (ie, in the presence of temporal irreversibility), the time-shifted correlation differs between the forward and time-reversed signals. Furthermore, it has been shown that pairwise interactions are sufficient to assess nonequilibrium dynamics in the brain using fMRI data (Geli et al. 2025).

**Figure 1:**
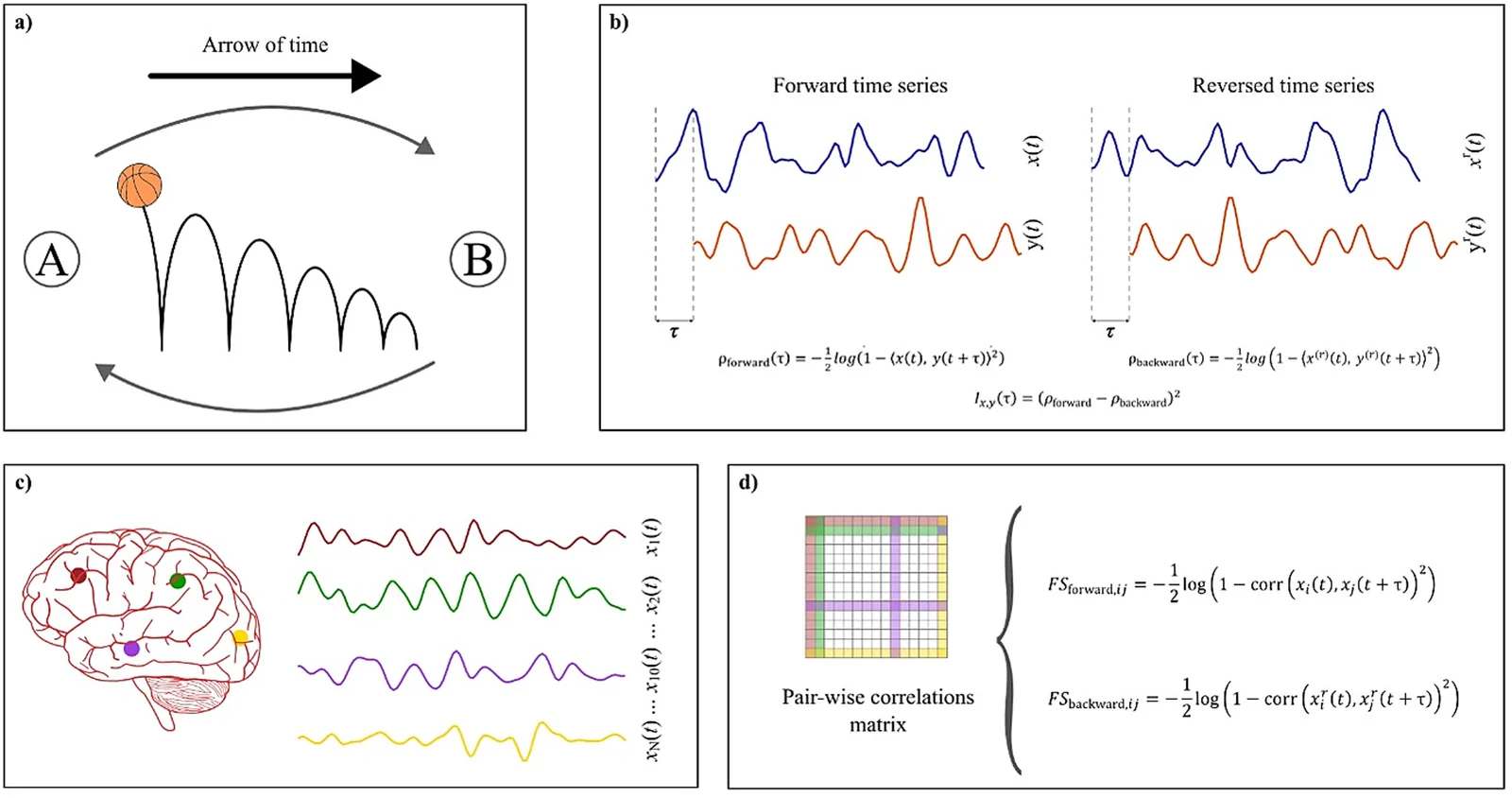
Overview of how to compute irreversibility: (a) the thermodynamic arrow of time is illustrated using a physical example (a bouncing basketball), where the forward trajectory and its artificially reversed counterpart are intuitively distinguishable. This illustrates the general principle of temporal irreversibility: The temporal evolution of an irreversible process differs from its time-reversed counterpart. (b) For a pair of brain time series, this difference cannot be assessed visually. The same principle is therefore quantified statistically by comparing lagged temporal dependencies in the original time series with those obtained after reversing the time series. Temporal irreversibility is defined from the difference between these forward and time-reversed lagged relationships. (c) We use multidimensional BOLD fMRI time-series data from the brain as our system. (d) From the multidimensional time-series data, we compute a matrix of pairwise time-shifted correlations for the forward signals (FS_forward_) and another for the artificially time-reversed signals (FS_backward_). The squared difference between these two matrices is averaged to obtain a global measure of temporal irreversibility.

Consider a multidimensional signal $\boldsymbol{x}(t)$, such as the BOLD fMRI data used in this study (Fig. 1c), where each time series corresponds to a brain region defined by the Schaefer 100-parcel cortical atlas, and *t* denotes the discrete time index of the sampled BOLD signal. The time series of parcels *i* and *j*, ${x}_i(t)$ and ${x}_j(t)$, and their respective reversed versions ${x_i}^{(r)}(t)$ and ${x_j}^{(r)}(t)$ are selected. The temporal dependency between them is quantified through the time-shifted correlation at a given temporal shift *τ* (Fig. 1b). That is, we compute a single Pearson correlation coefficient between ${x}_i(t)$ and the time-shifted signal ${x}_j\left(t+\tau \right)$ across all overlapping time points. We further applied a logarithmic transformation, to express these values as mutual information, ensuring well-behaved positive values.

In the forward case, this is defined as:


(1) \begin{eqnarray*} {\mathrm{FS}}_{\mathrm{forward},\kern0.36em ij}\left(\tau \right)=-\frac{1}{2}\log \left(1-{\left\langle{x}_i(t),{x}_j\left(t+\tau \right)\right\rangle}^2\right) \end{eqnarray*}

And in the backwards case:


(2) \begin{eqnarray*} {\mathrm{FS}}_{\mathrm{backward},\kern0.36em ij}\left(\tau \right)=-\frac{1}{2}\log \left(1-{\left\langle{x_i}^{(r)}(t),{x_j}^{(r)}\left(t+\tau \right)\right\rangle}^2\right) \end{eqnarray*}

where $\left\langle{x}_i(t),{x}_j\left(t+\tau \right)\right\rangle$ denotes the Pearson correlation coefficient computed between the time series of region *i* and the time-shifted time series of region *j*. Evaluating Eqs. (1) and (2) for every pair of brain regions (*i*, *j*) this yields two *N× N* matrices: one for the forward time series and one for the reversed time series (Fig. 1d), whose (*i, j*) element is $\mathrm{F}{\mathrm{S}}_{ij}\left(\tau \right)$. Then, pair-wise irreversibility is then defined as the quadratic distance between the forward and backward time-shifted correlations:


(3) \begin{eqnarray*} {I}_{ij}\left(\tau \right)={\left({\mathrm{FS}}_{\mathrm{forward}, ij}\left(\tau \right)-{\mathrm{FS}}_{\mathrm{backward}, ij}\left(\tau \right)\right)}^2 \end{eqnarray*}

The global level of irreversibility for each subject is obtained by averaging across all elements of $I\left(\tau \right)$. The level of irreversibility for a specific subdivision of the brain parcellation, such as a resting-state network (Yeo et al. 2011), is obtained by averaging across all node pairs belonging to that network.

Node-level irreversibility (ie, the irreversibility of a single ROI in the parcellation) is computed as follows, considering the *n*th node:


(4) \begin{eqnarray*} {I}_n=\frac{\sum_{i=1}^N{I}_{i,j=n}\left(\tau \right)}{N}+\frac{\sum_{j=1}^N{I}_{i=n,\kern0.5em j}\left(\tau \right)}{N} \end{eqnarray*}

We selected a time shift value of *τ =1* (2 s), corresponding to one TR, because it is the shortest temporal dependency resolvable by our acquisition, thereby minimizing the temporal separation while still testing temporal asymmetry. Parcel-wise BOLD signal autocorrelation across temporal lags confirmed the expected strongest temporal dependence at *τ* = 1, consistent with the slow temporal characteristics of the BOLD signal (Supplementary Fig. S2). Larger values of *τ* become increasingly harder to interpret because the BOLD signal increasingly reflects the sluggish hemodynamic response and regional variability in the hemodynamic response function. To assess the robustness of the findings, all analyses were repeated using *τ* = 2, 3, and 4 as sensitivity analyses.

### Statistical analysis

Group characteristics and cognitive variables were examined for normality and homogeneity of variance prior to analysis. Statistical analyses were performed using IBM SPSS Statistics, version 29 (IBM Corp., Armonk, NY, USA). Depending on data distribution, group differences were assessed using independent samples *t*-tests or Mann–Whitney *U* tests. All tests were two-tailed, with a significance threshold of *P* < 0.05.

Monte Carlo permutation testing was performed to assess group differences in temporal irreversibility at both the network and node levels, providing nonparametric inference robust to distributional assumptions. For the node-level analysis, permutation tests were performed independently for each of the 100 cortical parcels. Mean differences between groups (Controls − EPT) were calculated to indicate the direction of the effect. Correction for multiple comparisons was performed using the false discovery rate (FDR) procedure according to Benjamini and Hochberg (1995). FDR correction was also applied separately to the node-level, network-level, and correlation analyses.

For significant group comparisons, effect sizes (Cohen’s *d*), and corresponding 95% confidence intervals were calculated based on group sample sizes. To formally determine whether the association between temporal irreversibility and fluid reasoning differed between EPT and term-born children, linear regression models were fitted including group, irreversibility, and a Group × Irreversibility interaction term. Separate models were performed for global irreversibility and salience network irreversibility. Irreversibility measures were mean-centered before constructing the interaction term to improve coefficient interpretability and reduce multicollinearity.

## Results

### Demographics

Participant characteristics have been reported previously (Padilla et al. 2020). A total of 112 children (66 EPT, 46 term-born controls) underwent MRI at approximately10 years of age. Following exclusion of participants with brain abnormalities (7 EPT; 2 term-born) and low-quality MRI data, mainly related to motion (26 EPT; 18 term-born), the final sample included 33 EPT children and 26 term-born controls (Table 1 and Supplementary Fig. S1). Neonatal characteristics of the EPT group are presented in Supplementary Table S2. Dropout analyses comparing included and excluded EPT children revealed no significant differences (Supplementary Table S3). The two groups also did not differ in motion parameters (Supplementary Table S4).

**Table 1:** Characteristics of the groups.

Characteristics	EPT	Term	Statistic (*P*)
	*N* = 33	*N* = 26	
**Perinatal data**			
Mean gestational age in weeks (SD)	25.70 (0.95)	39.93 (1.12)	*t* = −52.8 (<0.001)
range	23.5–26.6	37.3–41.5	
Mean birth weight in grams (SD)	856.2 (173.4)	3663 (421.0)	*t* = −31.8 (<0.001)
range	550–1161	2875–4720	
Gender (boy/girl)	13/20	14/12	Fisher's test (0.17)
Mean age at MRI in years (SD)	10.06 (0.82)	9.92 (0.88)	*t* = 0.65 (0.52)
range	9.0–11.5	8.0–11.8	
**WISC-Fifth Edition index**	**EPT *N* = 29**	**Term *N* = 21**	***P* value**
	**Mean (SD)**	**Mean (SD)**	
Mean age at WISC-V in years (SD)	12.14 (0.22)	12.10 (0.23)	*t* = 1.97 (0.051)
Range	11.83–12.83	11.58–12.42	
Verbal comprehension	100.70 (16.14)	118.76 (16.45)	*t* = −3.71 (<0.001)
Visuospatial	91.50 (14.97)	102.57 (14.60)	*t* = −2.50 (0.016)
Fluid reasoning	94.79 (11.08)	106.85 (10.48)	*t* = −3.73 (<0.001)
Working memory	90.12 (17.61)	101.38 (12.42)	*t* = −2.44 (0.019)
Processing speed	95 (15.99)	107.52 (13.66)	*t* = −2.80 (0.008)
Total IQ	94.29 (14.11)	112.42 (12.70)	*t* = −4.50 (<0.001)

EPT, extremely preterm group; SD, standard deviation; WISC-V, Wechsler Intelligence Scale for Children-Fifth Edition V; IQ, intelligence quotient.

### Cognitive assessments at 12 years of age

Cognitive assessments were available for 29 of the 33 EPT children and 21 of the 26 term-born controls, who were therefore included in the brain–behavior analyses. Children born EPT scored significantly lower across all WISC-V indices compared with term-born controls (Table 1). All group differences survived correction for multiple comparisons using the FDR procedure.

### Temporal irreversibility at 10 years of age

Temporal irreversibility was significantly lower at the global level in children born EPT compared with term-born controls (EPT: 1.321 ± 0.427 × 10^−3^; term: 1.588 ± 0.628 × 10^−3^; FDR-corrected *P* = 0.0096). At the network level, reduced irreversibility in the EPT group was observed in the default mode, control, salience/ventral attention, and dorsal attention networks after FDR correction (Figs. 2 and 3; Supplementary Table S5). No significant group differences were observed in the visual, somatomotor, or limbic networks. No network showed higher temporal irreversibility in the EPT group compared with term-born controls.

**Figure 2:**
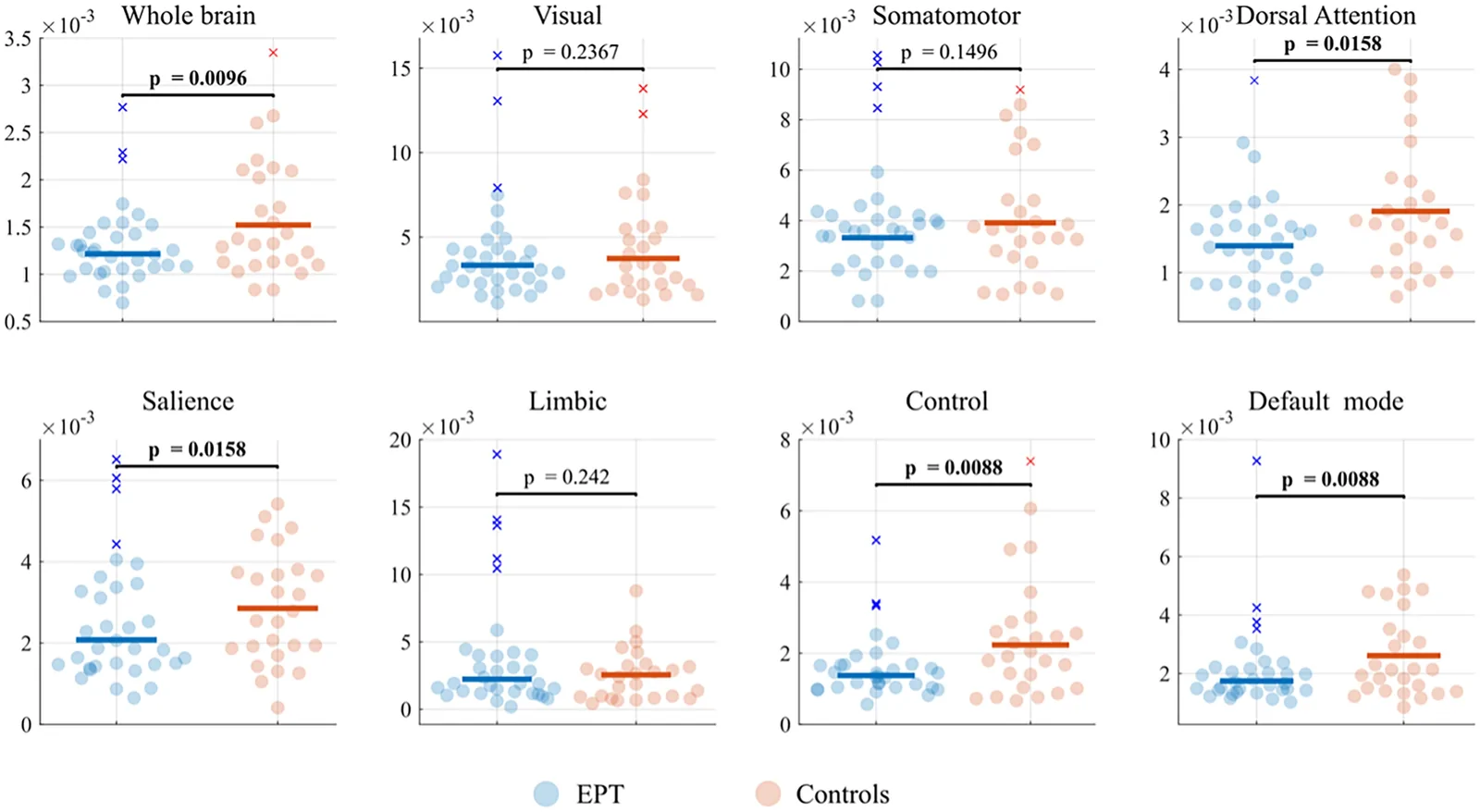
Temporal irreversibility in the whole brain and seven resting state networks. Group comparisons of temporal irreversibility at 10 years of age for the whole brain and seven large-scale functional networks (visual, somatomotor, dorsal attention, salience, limbic, control, and default mode). Blue indicates children born EPT (*N* = 33), and orange denotes term-born controls (*N* = 26). Horizontal lines indicate group medians. Group differences were assessed using Monte Carlo permutation testing on group mean differences (two-tailed), with exact *P* values shown in the results and Supplementary Table 5. *P* values were corrected for multiple comparisons using the false discovery rate procedure at α = 0.05. Blue and orange × symbols indicate values exceeding 1.5 times the interquartile range.

**Figure 3:**
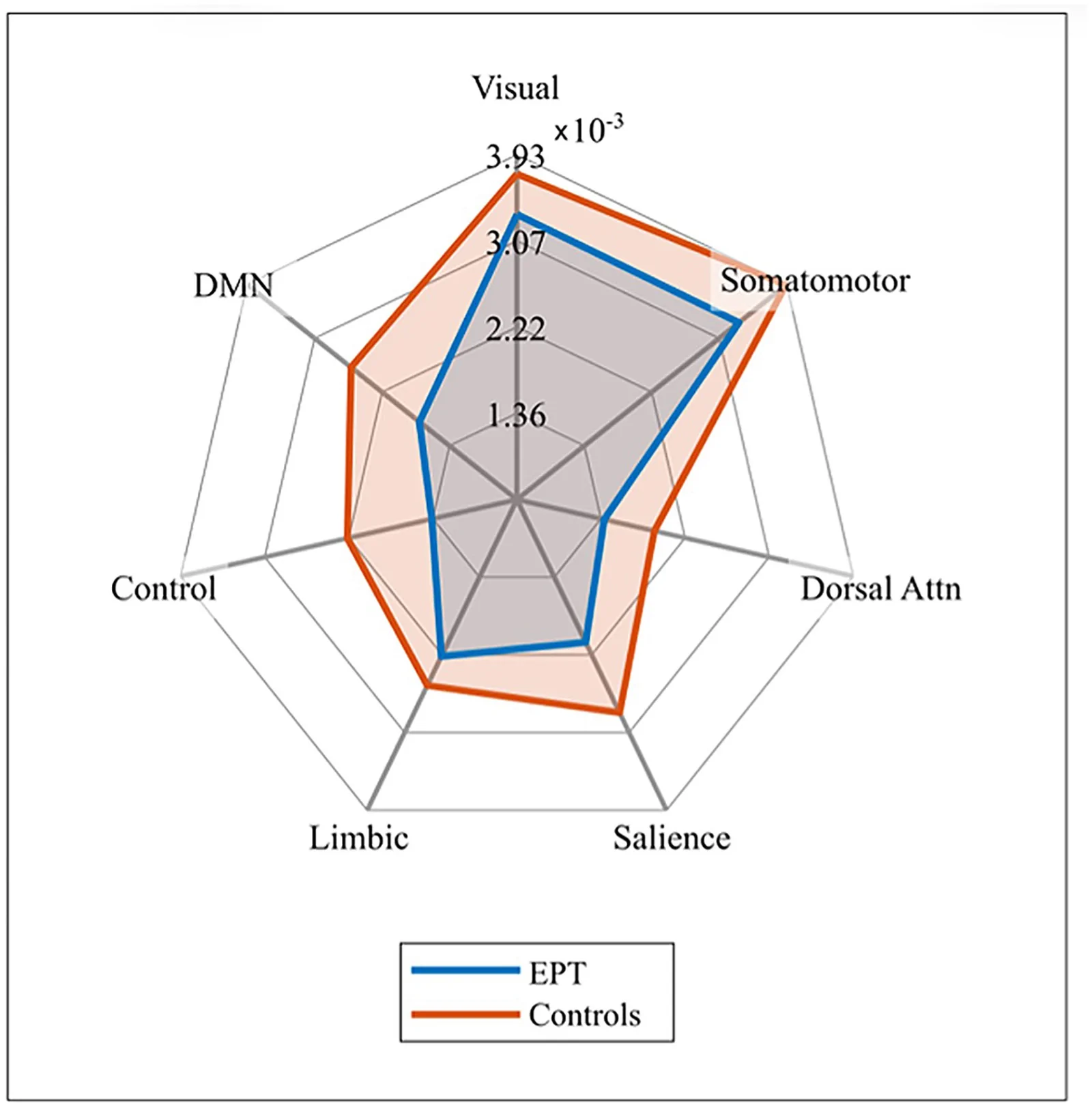
Network profile of temporal irreversibility. Mean temporal irreversibility across the seven functional networks in children born EPT and term-born controls. Values represent group means (×10^−3^). Although statistically significant differences were observed, only in the default mode, control, salience/ventral attention, and dorsal attention networks (Fig. 2), the radar plot illustrates the comparatively smaller alterations observed in the visual and somatomotor networks in children born EPT.

The results remained unchanged after exclusion of outliers (>1.5× interquartile range; Supplementary Tables S6 and S7). Effect sizes were generally small to moderate (Cohen’s *d* ≈ 0.3–0.6), with 95% confidence intervals reported in Supplementary Table S8. No significant sex differences in global or network-level temporal irreversibility were observed after FDR correction across the eight measures. The parcel-wise BOLD signal autocorrelation showed similar profiles in children born EPT and term-born controls, supporting the selection of *τ* = 1 for the primary analyses (Supplementary Fig. S2). Sensitivity analyses using temporal shifts of *τ* = 2–4 showed a similar overall pattern of group differences, although statistical significance progressively decreased with increasing *τ* (Supplementary Fig. S3). We additionally calculated the hierarchy metric proposed by (Deco et al. 2022), defined as the spatial variability of temporal irreversibility across brain regions. Although it showed a similar directional trend to mean temporal irreversibility, no network remained significant after FDR correction (Supplementary Fig. S4).

### Node-level differences in temporal irreversibility

Monte Carlo permutation testing identified 24 cortical parcels with lower temporal irreversibility in children born EPT at the uncorrected permutation threshold (permutation *P* < 0.05), predominantly within the default mode, control, dorsal attention, and salience/ventral attention networks (Supplementary Table S9). After FDR correction across the 100 cortical parcels, two nodes remained significant: the left precuneus within the control network and the right dorsomedial prefrontal cortex within the default mode network (both FDR-corrected *P* = 0.04995). No cortical parcel showed significantly higher temporal irreversibility in the EPT group before or after FDR correction, although several visual and somatomotor parcels showed numerically higher values.

### Associations between temporal irreversibility and cognitive performance

Within the EPT group, fluid reasoning was negatively associated with global temporal irreversibility (*r* = −0.504, *P* = 0.007) and salience network temporal irreversibility (*r* = −0.459, *P* = 0.016) (Table 2). These associations remained significant after correction for multiple comparisons. No other significant correlations were observed. In the term-born group, global temporal irreversibility was negatively correlated with fluid reasoning and processing speed (*r* = −0.443, *P* = 0.044 for both). These associations did not survive correction for multiple comparisons (Table 2). The interaction between group and global temporal irreversibility was statistically significant (*B* = 19,675.62 (95% CI 1,911.60–37,439.64), *P* = 0.031). In contrast, the interaction between group and salience network temporal irreversibility was not significant (*B* = 2,723.45 (95% CI −2,212.22 to 7,659.11), *P* = 0.272).

**Table 2:** Correlations between brain data and cognitive performance at 12 years.

Extremely preterm children					
Cognitive indexes					
Network	Fluid reasoning	Working memory	Processing speed	Verbal comprehension	Visual spatial
	*r, P*	*r, P*	*r, P*	*r, P*	*r, P*
Global	**−0.504 (0.007)**	−0.092 (0.648)	−0.135 (0.502)	0.038 (0.852)	−0.263 (0.186)
Default mode	−0.104 (0.599)	0.076 (0.700)	−0.104 (0.599)	−0.062 (0.753)	−0.153 (0.437)
Salience	**−0.459 (0.016)**	−0.222 (0.265)	−0.267 (0.179)	0.077 (0.702)	−0.197 (0.325)
Dorsal attention	−0.002 (0.990)	−0.029 (0.882)	−0.002 (0.990)	−0.047 (0.813)	−0.017 (0.931)
Control	0.057 (0.783)	−0.090 (0.661)	0.173 (0.398)	0.239, 0.240	−0.076 (0.711)
**Term-born children**					
**Cognitive indexes**					
**Network**	**Fluid reasoning**	**Working memory**	**Processing speed**	**Verbal comprehension**	**Visual spatial**
	***r, P***	***r, P***	***r, P***	***r, P***	***r, P***
Global	−0.443 (0.044)^a^	−0.120 (0.606)	−0.443 (0.044)^a^	0.333 (0.140)	−0.278, 0.222
Default mode	−0.029 (0.904)	−0.127 (0.593)	−0.417 (0.067)	0.066 (0.781)	0.027 (0.908)
Salience	−0.162 (0.484)	0.015 (0.948)	−0.064 (0.783)	0.094 (0.686)	−0.247 (0.279)
Dorsal attention	0.215 (0.350)	0.165 (0.476)	0.083 (0.721)	0.404 (0.070)	0.044 (0.849)
Control	−0.098 (0.673)	0.073 (0.755)	0.175 (0.447)	0.119 (0.607)	−0.228 (0.319)

^a^ *P*–value < 0.05. Bold values remained significant after correction for multiple comparisons (false discovery rate method).

## Discussion

This study provides evidence that extreme prematurity is associated with persistent alterations in the temporal organization of brain activity at 10 years of age. Children born EPT showed reduced temporal irreversibility at the global level and across higher-order association networks, particularly the default mode, control, salience, and dorsal attention networks, whereas no significant alterations were observed in the visual or somatomotor networks. At the node level, the findings were consistent with the network-level results, with reduced temporal irreversibility concentrated within higher-order association regions and two cortical parcels remaining significant after FDR correction. In addition, the association between global temporal irreversibility and fluid reasoning differed significantly between EPT and term-born children, highlighting altered brain–behavior relationships following extreme prematurity. Together, these findings suggest persistent alterations in the large-scale organization of brain dynamics that are associated with later fluid reasoning.

These results extend our previous findings in the same cohort demonstrating reduced intrinsic ignition and metastability (Padilla et al. 2020; Padilla et al. 2023), indicating convergent alterations across complementary measures of large-scale brain dynamics following extreme prematurity. Because healthy brain function relies on nonequilibrium dynamics, the functional implications of these alterations require further investigation.

Reduced temporal irreversibility was most pronounced within higher-order association networks, including the default mode, control, salience, and dorsal attention networks. Although these differences remained significant after permutation testing and correction for multiple comparisons, the corresponding effect sizes were generally small to moderate and associated with relatively wide confidence intervals, indicating that the magnitude of the observed effects should be interpreted with caution despite robust evidence for group differences. This likely reflects the modest sample size, which limits the precision of the estimated effect sizes despite consistent nonparametric evidence for between-group differences. Taken together, these findings suggest distributed alterations in large-scale brain dynamics rather than focal abnormalities. The node-level analyses further supported this pattern by localizing the most robust reductions in temporal irreversibility to higher-order association regions. These findings highlight the triple-network system (Menon 2011; Deco et al. 2023), as a particularly vulnerable substrate following extreme prematurity (Tokariev et al. 2025), potentially reflecting delayed maturation or inefficient large-scale network integration (Derbie et al. 2025; Mousley et al. 2025; Lean et al. 2026). Our findings are also consistent with recent evidence of reduced segregation between the default mode, frontoparietal, salience, and dorsal attention networks in school-aged children born preterm (Urriola et al. 2025), supporting the notion that these higher-order association networks represent persistent developmental vulnerabilities following preterm birth.

In contrast, although several visual and somatomotor parcels showed numerically higher temporal irreversibility in children born EPT, none reached statistical significance. Although these differences did not reach statistical significance, this descriptive spatial pattern is consistent with our previous studies showing relatively preserved or increased intrinsic ignition and metastability within lower-order sensorimotor networks (Padilla et al. 2020; Padilla et al. 2023). Within the hierarchical framework of brain maturation, in which early maturing sensory networks progressively give way to higher-order control networks (Gao 2025), these converging findings support the notion that higher-order association networks are preferentially affected following extreme prematurity, whereas primary sensorimotor systems appear relatively preserved.

Converging evidence from neonatal fMRI (Ma et al. 2020; França et al. 2024; Mella et al. 2024; Damera et al. 2025), and EEG (Adibpour et al. 2025), indicates that alterations in brain dynamics are already present at term-equivalent age in preterm infants, including atypical dynamic connectivity, slower temporal organization, and reduced variability. Our findings extend these observations by demonstrating reduced temporal irreversibility persist into later childhood, predominantly affecting higher-order association networks. This is consistent with a perturbation of a critical in utero phase of spatially and temporally structured endogenous activity (Yrjölä et al. 2022; Stevenson et al. 2025), with developmental consequences evident during the neonatal period (França et al. 2024), and extending into later childhood (Padilla et al. 2020; Padilla et al. 2023; Siffredi et al. 2023; Tokariev et al. 2025).

Notably, the negative association between higher temporal irreversibility, both at the global level and within the salience network, and fluid reasoning was unexpected, as pathological conditions have typically been characterized by reduced temporal irreversibility (Cruzat et al. 2023; Guzmán et al. 2023). Importantly, formal interaction analyses demonstrated that the association between global temporal irreversibility and fluid reasoning differed significantly between EPT and term-born children, suggesting that this relationship is not simply a common association observed across both groups. These findings suggest that group-level reductions in temporal irreversibility and inter-individual variability within the EPT group may reflect distinct neurobiological processes rather than opposite manifestations of the same mechanism. Importantly, the direction of the association between temporal irreversibility and cognitive performance should not be assumed a priori and requires replication in independent cohorts.

Although a similar negative association was observed within the salience network, the Group × Salience interaction was not significant and should therefore be interpreted with caution. Given the stablished role of the salience network, in rapid switching between large-scale brain systems (Menon and Uddin 2010), alterations in its temporal dynamics may be relevant for cognitive function. The underlying neurobiological mechanisms require confirmation in independent cohorts and mechanistic studies.

Extreme prematurity may be associated with a global reduction in directional, time-asymmetric dynamics, with preferential alterations in higher-order association networks. In this context, higher temporal irreversibility should not necessarily be interpreted as reflecting healthier brain dynamics. Instead, the present findings suggest that the relationship between temporal irreversibility and fluid reasoning is more complex and may differ following extreme prematurity. The direction and biological basis of this relationship cannot be inferred from the present study and remain to be established.

These findings have important translational implications, given that altered large-scale brain dynamics have been implicated in neurodevelopmental and psychiatric disorders such as schizophrenia (Watanabe and Yamasue 2025). Cognitive and neurodevelopmental alterations following extreme prematurity are well established as developmental pathways associated with later mental health difficulties; in this context, the preferential involvement of higher-order association networks observed here overlap with features previously implicated in neurodevelopmental and psychiatric disorders, supporting the relevance of these findings to biological mechanisms of psychiatric vulnerability. Identifying altered large-scale brain dynamics in children born EPT may help inform future studies of developmentally targeted interventions. The preferential involvement of higher-order association networks, together with the relative preservation of visual and somatomotor networks, suggests that the developmental trajectories of these systems warrant further investigation as potential targets in future intervention studies. Optimization of the perinatal environment through brain-centered care practice (Volpe 2019; Molloy et al. 2024), together with behavioral, cognitive, and emerging noninvasive neuromodulatory approaches (To et al. 2018; Zhang et al. 2023; Zhong et al. 2025), may help promote more adaptive network dynamics and improve long-term cognitive outcomes.

Several limitations should be acknowledged. Although the sample size was relatively small, the cohort is uniquely characterized by detailed longitudinal clinical, neuroimaging, and cognitive data, and rigorous quality assurance was essential for obtaining reliable imaging measures. Resting-state fMRI was acquired with the participants’ eyes closed and without objective monitoring of vigilance (eg, eye tracking or EEG). Although all children were scanned without sedation, instructed to remain awake, and continuously supervised throughout the examination, subtle fluctuations in arousal cannot be completely excluded. Furthermore, temporal irreversibility was estimated from preprocessed BOLD signals, and preprocessing choices, including temporal filtering, may influence its absolute magnitude. The limited temporal resolution of BOLD fMRI constrains the interpretation of temporal dependencies at longer lags. However, sensitivity analyses using multiple temporal shifts showed a similar overall pattern of results, supporting the robustness of the main findings. Identical preprocessing was applied to all participants, making it unlikely that these factors account for the observed between-group differences. Finally, the cross-sectional design precludes conclusions about developmental trajectories, and the approximately 2-year interval between MRI acquisition and cognitive assessment limits the interpretation of the observed brain-behavior associations. Longitudinal and intervention studies in larger cohorts are needed to determine how brain dynamics evolve following extreme prematurity and to support translation into clinical practice.

In conclusion, extreme prematurity is associated with persistent alterations in brain dynamics at 10 years of age, characterized by reduced temporal irreversibility at the global level and across higher-order association networks. Node-level analyses further supported this pattern, with the most robust alterations localized to higher-order association regions. Importantly, the relationship between global temporal irreversibility and fluid reasoning differed between children born EPT and term-born controls, suggesting that the functional significance of temporal irreversibility is altered following extreme prematurity. Together, these findings indicate that extreme prematurity leads to persistent alterations in the large-scale brain dynamics. Temporal irreversibility may therefore provide information about large-scale dynamic brain organization following extreme prematurity, while its predictive and clinical relevance will require evaluation in independent longitudinal cohorts.

## Supplementary Material

Supplementary_Material_bhag174

## References

[ref1] Adibpour P et al. 2025. Characterizing the temporal dynamics and maturation of brain activity during sleep: an EEG microstate study in preterm and full-term infants. Imaging Neurosci. 3. 10.1162/imag_a_00450. PMC12319786 40800944

[ref2] Behboodi S et al. 2025. Trends over time in cognitive outcomes of children born very preterm: a systematic review and meta-analysis. JAMA Pediatr. 179:1074–1081. 10.1001/jamapediatrics.2025.2221. PMC12305443 40720136

[ref3] Benjamini Y, Hochberg Y. 1995. Controlling the false discovery rate: a practical and powerful approach to multiple testing. J R Stat Soc. 57:289–300. 10.1111/j.2517-6161.1995.tb02031.x.

[ref4] Cara C et al. 2025. Prenatal brain connectivity and postnatal language: how familial risk and prenatal speech exposure shape early language skills. Sci Rep. 15:33281. 10.1038/s41598-025-17531-y. PMC12475424 41006557

[ref5] Cruzat J et al. 2023. Temporal irreversibility of large-scale brain dynamics in Alzheimer’s disease. J Neurosci. 43:1643–1656. 10.1523/JNEUROSCI.1312-22.2022. PMC10008060 36732071

[ref6] Damera SR et al. 2025. Altered brain state dynamics between preterm and term-born infants. Imaging Neurosci. 3:IMAG.a.65. 10.1162/IMAG.a.65. PMC12330851 40800740

[ref7] Deco G, Sanz Perl Y, Bocaccio H, Tagliazucchi E, Kringelbach ML. 2022. The INSIDEOUT framework provides precise signatures of the balance of intrinsic and extrinsic dynamics in brain states. Commun Biol. 5:572. 10.1038/s42003-022-03505-7. PMC9187708 35688893

[ref8] Deco G et al. 2023. The arrow of time of brain signals in cognition: potential intriguing role of parts of the default mode network. Netw Neurosci. 7:966–998. 10.1162/netn_a_00300. PMC10473271 37781151

[ref9] Derbie AY et al. 2025. Early life brain network connectivity antecedents of executive function in children born preterm. Commun Biol. 8:345. 10.1038/s42003-025-07745-1. PMC11873160 40025105

[ref10] EXPRESS Group et al. 2009. One-year survival of extremely preterm infants after active perinatal care in Sweden. JAMA. 301:2225–2233. 10.1001/jama.2009.771. 19491184

[ref11] França LGS et al. 2024. Neonatal brain dynamic functional connectivity in term and preterm infants and its association with early childhood neurodevelopment. Nat Commun. 15:16. 10.1038/s41467-023-44050-z. PMC10853532 38331941

[ref12] Gao W . 2025. A hierarchical model of early brain functional network development. Trends Cogn Sci. 29:855–868. 10.1016/j.tics.2025.04.001. PMC12353820 40335413

[ref13] Geli SM, Lynn CW, Kringelbach ML, Deco G, Sanz Perl Y. 2025. Non-equilibrium whole-brain dynamics arise from pairwise interactions. Cell Rep Phys Sci. 6:102464. 10.1016/j.xcrp.2025.102464.

[ref14] Guzmán E et al. 2023. The lack of temporal brain dynamics asymmetry as a signature of impaired consciousness states. Interface Focus. 13:20220086. 10.1098/rsfs.2022.0086. PMC10102727 37065259

[ref15] Kostović I, Sedmak G, Judaš M. 2019. Neural histology and neurogenesis of the human fetal and infant brain. NeuroImage. 188:743–773. 10.1016/j.neuroimage.2018.12.043. 30594683

[ref16] Kringelbach ML, Perl YS, Tagliazucchi E, Deco G. 2023. Toward naturalistic neuroscience: mechanisms underlying the flattening of brain hierarchy in movie-watching compared to rest and task. Sci Adv. 9:eade6049. 10.1126/sciadv.ade6049. PMC9839335 36638163

[ref17] Kringelbach ML, Sanz Perl Y, Deco G. 2024. The thermodynamics of mind. Trends Cogn Sci. 28:568–581. 10.1016/j.tics.2024.03.009. 38677884

[ref18] Kvanta H et al. 2024. Language performance and brain volumes, asymmetry, and cortical thickness in children born extremely preterm. Pediatr Res. 95:1070–1079. 10.1038/s41390-023-02871-0. PMC10920199 37923870

[ref19] Lean RE et al. 2026. Prenatal adversity and neonatal brain connectivity relate to emerging executive function at age 2 years. J Am Acad Child Adolesc Psychiatry. 65:564–576. 10.1016/j.jaac.2025.04.016. PMC12284968 40334775

[ref20] Li Q et al. 2026. Deciphering multiway multiscale brain network connectivity from birth to 6 months. Commun Biol [published online ahead of print]. accessed 2026 Feb 1 9:271. 10.1038/s42003-026-09549-3. PMC12917168 41545740

[ref21] Ma X, Wu X, Shi Y. 2020. Changes of dynamic functional connectivity associated with maturity in late preterm infants. Front Pediatr. 8:412. 10.3389/fped.2020.00412. PMC7390889 32793532

[ref22] Mella AE, Vanderwal T, Miller SP, Weber AM. 2024. Temporal complexity of the BOLD-signal in preterm versus term infants. Cereb Cortex. 34:bhae426. 10.1093/cercor/bhae426. PMC11586500 39582376

[ref23] Menon V . 2011. Large-scale brain networks and psychopathology: a unifying triple network model. Trends Cogn Sci. 15:483–506. 10.1016/j.tics.2011.08.003. 21908230

[ref24] Menon V, Uddin LQ. 2010. Saliency, switching, attention and control: a network model of insula function. Brain Struct Funct. 214:655–667. 10.1007/s00429-010-0262-0. PMC2899886 20512370

[ref25] Molloy EJ et al. 2024. Neuroprotective therapies in the NICU in preterm infants: present and future (neonatal neurocritical care series). Pediatr Res. 95:1224–1236. 10.1038/s41390-023-02895-6. PMC11035150 38114609

[ref26] Mousley A, Akarca D, Astle DE. 2025. Premature birth changes wiring constraints in neonatal structural brain networks. Nat Commun. 16:490. 10.1038/s41467-024-55178-x. PMC11711473 39779695

[ref27] Padilla N et al. 2020. Breakdown of whole-brain dynamics in preterm-born children. Cereb Cortex. 30:1159–1170. 10.1093/cercor/bhz156. PMC7132942 31504269

[ref28] Padilla N et al. 2023. Disrupted resting-sate brain network dynamics in children born extremely preterm. Cereb Cortex. 33:8101–8109. 10.1093/cercor/bhad101. PMC10321088 37083266

[ref29] Pascoe L, Burnett AC, Anderson PJ. 2021. Cognitive and academic outcomes of children born extremely preterm. Semin Perinatol. 45:151480. 10.1016/j.semperi.2021.151480. 34656363

[ref30] Sadeghi F et al. 2025. The arrow of time in Parkinson’s disease. NeuroImage Clin. 47:103834. 10.1016/j.nicl.2025.103834. PMC12269992 40592209

[ref31] Schaefer A et al. 2018. Local-global parcellation of the human cerebral cortex from intrinsic functional connectivity MRI. Cereb Cortex. 28:3095–3114. 10.1093/cercor/bhx179. PMC6095216 28981612

[ref32] Serenius F et al. 2013. Neurodevelopmental outcome in extremely preterm infants at 2.5 years after active perinatal Care in Sweden. JAMA. 309:1810–1820. 10.1001/jama.2013.3786. 23632725

[ref33] Serenius F et al. 2023. Neurobehavioral symptoms in children born extremely preterm: a Swedish National Study. Acta Paediatr. 112:2387–2399. 10.1111/apa.16942. 37551108

[ref34] Siffredi V et al. 2023. Large-scale brain network dynamics in very preterm children and relationship with socio-emotional outcomes: an exploratory study. Pediatr Res. 93:2072–2080. 10.1038/s41390-022-02342-y. PMC10313511 36329223

[ref35] Skiöld B et al. 2010. White matter changes in extremely preterm infants, a population-based diffusion tensor imaging study. Acta Paediatr. 99:842–849. 10.1111/j.1651-2227.2009.01634.x. 20132144

[ref36] Stevenson NJ, Iyer K, Tokariev A, Roberts JA, Vanhatalo S. 2025. Maturation of neuronal activity in the human cortex exhibits robust spatial gradients across the birth transition. J Neurosci. 45:e0610252025. 10.1523/JNEUROSCI.0610-25.2025. PMC12509498 40883001

[ref37] To WT, De Ridder D, Hart J Jr, Vanneste S. 2018. Changing brain networks through non-invasive neuromodulation. Front Hum Neurosci. 12:128. 10.3389/fnhum.2018.00128. PMC5908883 29706876

[ref38] Tokariev M et al. 2025. Imprints of extreme prematurity on functional brain networks in school-aged children and adolescents. NeuroImage. 320:121447. 10.1016/j.neuroimage.2025.121447. 40930409

[ref39] Twilhaar ES, Wolke D. 2025. Fostering positive mental health outcomes in vulnerable children: pathways to resilience after preterm birth. J Child Psychol Psychiatry. 66:1860–1874. 10.1111/jcpp.70002. PMC12626177 40665809

[ref40] Urriola J et al. 2025. Altered functional brain organisation in preterm children: motor task and resting-state fMRI findings at six years. Neuroimage Clin. 48:103906. 10.1016/j.nicl.2025.103906. PMC12671035 41265076

[ref41] Volpe JJ . 2019. Dysmaturation of premature brain: importance, cellular mechanisms, and potential interventions. Pediatr Neurol. 95:42–66. 10.1016/j.pediatrneurol.2019.02.016. 30975474

[ref42] Watanabe T, Yamasue H. 2025. Noninvasive reduction of neural rigidity alters autistic behaviors in humans. Nat Neurosci. 28:1348–1360. 10.1038/s41593-025-01961-y. PMC12148933 40481227

[ref43] Yeo BT et al. 2011. The organization of the human cerebral cortex estimated by intrinsic functional connectivity. J Neurophysiol. 106:1125–1165. 10.1152/jn.00338.2011. PMC3174820 21653723

[ref44] Yrjölä P, Stjerna S, Palva JM, Vanhatalo S, Tokariev A. 2022. Phase-based cortical synchrony is affected by prematurity. Cereb Cortex. 32:2265–2276. 10.1093/cercor/bhab357. PMC9113310 34668522

[ref45] Zhang J et al. 2023. Reducing default mode network connectivity with mindfulness-based fMRI neurofeedback: a pilot study among adolescents with affective disorder history. Mol Psychiatry. 28:2540–2548. 10.1038/s41380-023-02032-z. PMC10611577 36991135

[ref46] Zhong M et al. 2025. Safety and effectiveness of non-invasive brain stimulation on mobility and balance function in children with cerebral palsy: a systematic review and meta-analysis. J Neuroeng Rehabil. 22:111. 10.1186/s12984-025-01619-7. PMC12087172 40383797

