# Supplementary material for: Temporal brain dynamics and fluid reasoning after extreme prematurity
Source: Cereb Cortex. 2026 Oct 19;36(10):bhag174. doi: 10.1093/cercor/bhag174 (PMC13677789; doi:10.1093/cercor/bhag174)
Supplement: Supplementary file 1 [file supplementary_material_bhag174.docx]

**Supplementary Material**

**Temporal brain dynamics and fluid reasoning after extreme prematurity**

Nelly Padilla^1*^, Anira Escrichs^2^, Elvira del Agua^2^, Morten Kringelbach^3,4^, Antonio Donaire^5,6^, Gustavo Deco^2,7,8,9^, Ulrika Åden^1,10,11^

1. Dept of Women’s and Children’s Health, Karolinska Institutet, Stockholm, S- 171 76 Sweden.

2. Computational Neuroscience Group, Center for Brain and Cognition, Department of Information and Communication Technologies, Universitat Pompeu Fabra, Barcelona, Spain.

3. Department of Psychiatry, University of Oxford, Warneford Hospital, Warneford Ln, Oxford OX3 7JX, Storbritannien, United Kingdom.

4. Center for Music in the Brain, Aarhus University Hospital Nørrebrogade 44, Building 10G, 4th and 5th floor, 8000 Aarhus C, Denmark

5. Department of Neurology, Insititute of Neuroscience, Hospital Clinic, Universidad de Barcelona and Institut D’investigacions Biomèdiques August Pi i Sunyer (IDIBAPS), 08036 Barcelona, Spain.

6. Biomedical Research Networking Center in Bioengineering, Biomaterials and Nanomedicine (CIBER-BBN), Barcelona, Spain.

7. School of Psychological Sciences, Monash University, Melbourne, Clayton VIC 3800, Australia.

8. Institució Catalana de la Recerca i Estudis Avancats (ICREA), Barcelona, Catalonia, Spain.

9. Department of Neuropsychology, Max Planck Institute for human Cognitive and Brain Sciences, Leipzig, Germany.

10. Dept of Neonatology, Karolinska University Hospital, Stockholm, S- 171 76 Sweden.

11. Department of Biomedical and Clinical Sciences. Linköping University, Linköping, Sweden.

**Corresponding author**

Nelly Padilla, MD, PhD, e-mail: [nelly.padilla@ki.se](mailto:nelly.padilla@ki.se)

Karolinska University Hospital. Department of Neonatology

Norrbacka, S3:03 Karolinska vägen 8

171 76 Stockholm, Sweden

Figure S1. Population

Figure S2. Empirical autocorrelation of the parcel-wise BOLD signal

Figure S3. Sensitivity analyses of temporal irreversibility using different temporal shifts (τ = 1–4)

Figure S4. Spatial variability of temporal irreversibility (hierarchy metric) across the whole brain and resting-state networks.

Table S1. Schaefer Atlas Node Definitions and Network Assignments

Table S2. Neonatal characteristics of the children born extremely preterm

Table S3. Drop-out analysis for children born EPT with and without high quality MRI

Table S4. Comparison of motion estimation values after AROMA

# Table S5. Temporal reversibility values in extremely preterm (EPT) and term-born groups: all data included

Table S6. Temporal reversibility values in extremely preterm (EPT) and term-born groups: excluding outliers

Table S7. Number of outliers on each group

Table S8. Effect sizes and 95% confidence intervals for group differences in temporal irreversibility.

Table S9. Nodes with higher temporal irreversibility in the EPT group

**Figure S1. Flow diagram showing participant recruitment, exclusions, MRI quality control, and the final samples included in the MRI and brain–behaviour analyses.**

**Figure S2. Empirical autocorrelation of the parcel-wise BOLD signal**

For each participant, autocorrelation functions were calculated for each cortical parcel and averaged across parcels before computing group means. The autocorrelation function showed an identical profile in children born extremely preterm and term-born controls, with a gradual decay across temporal lags. The strongest measurable temporal dependency occurred at **τ = 1**, supporting its selection for the primary analyses. **(a)** Average autocorrelation function across lags 0–20. **(b)** Expanded view of the first five temporal lags (τ = 0–5).

**Figure S3.** **Sensitivity analyses of temporal irreversibility using different temporal shifts (τ = 2–4)**

**Network-level temporal irreversibility across temporal shifts (τ = 1–4).** The overall pattern of group differences was preserved across temporal shifts, although statistical significance progressively decreased with increasing τ

**Figure S4. Spatial variability of temporal irreversibility (hierarchy metric) across the whole brain and resting-state networks.**

Spatial variability of temporal irreversibility (hierarchy metric) across the whole brain and resting-state networks, calculated according to Deco et al. (2022). Each point represents one participant and horizontal bars indicate group means. P-values were corrected for multiple comparisons using the Benjamini–Hochberg false discovery rate (FDR) (Benjamini & Hochberg, 1995). No network showed a significant group difference after FDR correction.

**Table S1. Schaefer Atlas Node Definitions and Network Assignments**

| **Name** | **Network Name** | **Full component name** |
| --- | --- | --- |
|  |  |  |
| 7Networks_RH_SalVentAttn_TempOccPar | salience / ventral attention | temporal occipital parietal |
| 7Networks_RH_SalVentAttn_PrC | salience / ventral attention | precentral |
| 7Networks_RH_SalVentAttn_FrOperIns | salience / ventral attention | frontal operculum insula |
| 7Networks_RH_SalVentAttn_PFCv | salience / ventral attention | ventral prefrontal cortex |
| 7Networks_RH_SalVentAttn_PFCl | salience / ventral attention | lateral prefrontal cortex |
| 7Networks_RH_SalVentAttn_Med | salience / ventral attention | medial |
| 7Networks_LH_SalVentAttn_ParOper | salience / ventral attention | parietal operculum |
| 7Networks_LH_SalVentAttn_TempOcc | salience / ventral attention | temporal occipital |
| 7Networks_LH_SalVentAttn_FrOperIns | salience / ventral attention | frontal operculum insula |
| 7Networks_LH_SalVentAttn_PFCl | salience / ventral attention | lateral prefrontal cortex |
| 7Networks_LH_SalVentAttn_Med | salience / ventral attention | medial |
| 7Networks_RH_DorsAttn_Post | dorsal attention | posterior |
| 7Networks_RH_DorsAttn_FEF | dorsal attention | frontal eye fields |
| 7Networks_RH_DorsAttn_PrCv | dorsal attention | precentral ventral |
| 7Networks_LH_DorsAttn_Post | dorsal attention | posterior |
| 7Networks_LH_DorsAttn_FEF | dorsal attention | frontal eye fields |
| 7Networks_LH_DorsAttn_PrCv | dorsal attention | precentral ventral |
| 7Networks_LH_Default_Par | default | parietal |
| 7Networks_LH_Default_Temp | default | temporal |
| 7Networks_LH_Default_PFC | default | prefrontal cortex |
| 7Networks_LH_Default_pCunPCC | default | precuneus posterior cingulate cortex |
| 7Networks_LH_Default_PHC | default | parahippocampal cortex |
| 7Networks_RH_Default_Par | default | parietal |
| 7Networks_RH_Default_Temp | default | temporal |
| 7Networks_RH_Default_PFCv | default | ventral prefrontal cortex |
| 7Networks_RH_Default_PFCdPFCm | default | dorsal and medial prefrontal cortex |
| 7Networks_RH_Default_pCunPCC | default | precuneus posterior cingulate cortex |
| 7Networks_RH_Limbic_OFC | limbic | orbital frontal cortex |
| 7Networks_RH_Limbic_TempPole | limbic | temporal pole |
| 7Networks_LH_Limbic_OFC | limbic | orbital frontal cortex |
| 7Networks_LH_Limbic_TempPole | limbic | temporal pole |
| 7Networks_LH_Cont_Par | control | parietal |
| 7Networks_LH_Cont_Temp | control | temporal |
| 7Networks_LH_Cont_PFCd | control | dorsal prefrontal cortex |
| 7Networks_LH_Cont_PFCl | control | lateral prefrontal cortex |
| 7Networks_LH_Cont_OFC | control | orbital frontal cortex |
| 7Networks_LH_Cont_PFCv | control | ventral prefrontal cortex |
| 7Networks_LH_Cont_pCun | control | precuneus |
| 7Networks_LH_Cont_Cing | control | cingulate |
| 7Networks_LH_Cont_PFCmp | control | medial posterior prefrontal cortex |
| 7Networks_RH_Cont_Par | control | parietal |
| 7Networks_RH_Cont_Temp | control | temporal |
| 7Networks_RH_Cont_PFCv | control | ventral prefrontal cortex |
| 7Networks_RH_Cont_PFCl | control | lateral prefrontal cortex |
| 7Networks_RH_Cont_pCun | control | precuneus |
| 7Networks_RH_Cont_Cing | control | cingulate |
| 7Networks_RH_Cont_PFCmp | control | medial posterior prefrontal cortex |
| 7Networks_RH_Vis | visual | visual |
| 7Networks_LH_Vis | visual | visual |
| 7Networks_RH_SomMot | somatomotor | somatomotor |
| 7Networks_LH_SomMot | somatomotor | somatomotor |

| **Table S2. Neonatal characteristics of the children born extremely preterm** | |  |
| --- | --- | --- |
|  |  |  |
| **Morbidity** | **EPT children** |  |
|  | N=33 |  |
|  |  |  |
| Antenatal steroids, n (%) | 29/32 (90.6) |  |
|  |  |  |
| Apgar score at 5 minutes, mode (range) | 8.0 (6-10) |  |
|  |  |  |
| Small for gestational age (birth weight for gestational age < -2SD), according to the Swedish growth curve | 2/33 (6.0) |  |
|  |  |  |
| Clinical Risk Index for Babies score, mean ± SD | 5.0 ± 3.7 |  |
|  |  |  |
| Days on ventilator, mean ± SD | 9.7 ±12.9 |  |
| range | (0-43) |  |
|  |  |  |
| Postnatal corticosteroid therapy, n (%) | 4/33 (12.1) |  |
|  |  |  |
| Bronchopulmonary dysplasia,* n (%) | 9/30 (30.0) |  |
|  |  |  |
| Sepsis (clinical diagnosis), n (%) | 22/32 (68.7) |  |
|  |  |  |
| Necrotizing enterocolitis, n (%) | 1/32 (3.12) |  |
|  |  |  |
|  |  |  |
| Patent ductus arteriosus treated with surgery, n (%) | 8/32 (25.0) |  |
|  |  |  |
| Patent ductus arteriosus treated with ibuprofen, n (%) | 19/32 (59.3) |  |
|  |  |  |
| Low-grade intraventricular hemorrhage, n (%) | 9/33 (27.7) |  |
|  |  |  |
| Mild white matter abnormalities (score 7-9), n (%) | 13/33 (39.3) |  |
|  |  |  |
| Retinopathy of prematurity, n (%) | 23/33 (69.6) |  |
|  |  |  |
| EPT, extremely preterm; SD, standard deviation; *Oxygen at 36 weeks of gestation. | |  |

**Table S3. Drop-out analysis of children born EPT with and without high quality MRI**

| **Neonatal characteristic** | **EPT included** | **EPT not included** | **Statistic (*p* value)** |
| --- | --- | --- | --- |
|  | **N=33** | **N=26** |  |
| Gestational age at birth, weeks (SD) | 25.7 (0.95) | 25.3 (1.0) | t=1.38 (0.17) |
| Weight at birth, g (SD) | 0.86 (0.17) | 0.81 (0.12) | t= 1.11 (0.26) |
| Antenatal steroids, n (%) | 29/32 (90.6) | 30/31 (96.8) | FET, 0.61 |
| Apgar score at 5 minutes, mode (range) | 8.13 (1.49) | 7.81 (1.75) | t=0.77 (0.74) |
| Small for gestational age* | 2/33 (6.1) | 2/32 (6.3) | FET, 1.00 |
| Clinical Risk Index for Babies score, mean  ± SD | 5.0 ± 3.7 | 5.13 ± 2.9 | t=-0.04 (0.96) |
| Days on ventilator, mean ± SD | 10 (13.08) | 12.90 (12.2) | -0.90 (0.37) |
| Postnatal corticosteroid therapy, n (%) | 4/32 (12.5) | 5/31 (16.1) | FET, 0.73 |
| Bronchopulmonary dysplasia, ** n (%) | 9/30 (30.0) | 15/31 (48.4) | FET, 0.19 |
| Sepsis (clinical diagnosis), n (%) | 22/32 (68.7) | 22/31 (71) | FET 1.00 |
| Necrotizing enterocolitis, n (%) | 2/32 (6.3) | 6/31 (19.4) | FET, 0.14 |
| Patent ductus arteriosus treated with surgery, n (%) | 8/32 (25.0) | 11/31 (25.5) | FET, 0.41 |
| Patent ductus arteriosus treated with ibuprofen, n (%) | 19/32 (59.4) | 22/29 (75.9) | FET, 0.18 |
| Low-grade intraventricular hemorrhage, n (%) | 9/30 (30.0) | 12/27 (44.4) | FET, 0.28 |
| Mild white matter abnormalities (score 7-9), n (%) | 12/29 (41.4) | 15/26 (57.7) | FET, 0.28 |
| Retinopathy of prematurity, n (%) | 23/29 (79.3) | 25/30 (83.3) | FET, 0.74 |
| **Characteristics at late childhood** |  |  |  |
| Age at MRI | 10.06 (0.82) | 9.84 (0.83) | t=1.43 (0.15) |
| Age at developmental assessment | 12.12 (0.23) | 12.22 (0.29) | t=-1.43 (0.15) |
| EPT, extremely preterm; SD, standard deviation; *Small for gestational age, < -2SD; **Oxygen at 36 weeks of gestation., FTE, Fisher’s Exact Test.  The dropout analysis included 59 children born EPT who remained eligible after exclusion of seven participants with brain abnormalities. Of these, 33 had high-quality resting-state fMRI data and were included in the analyses, whereas 26 were excluded because of insufficient MRI quality. Denominators vary across variables because of missing data. | | | |

**Table S4. Comparison of motion estimation values after AROMA**

| **Parameter** | **Preterm** | **Term** | **Statistic,** |
| --- | --- | --- | --- |
|  | N=33 | N=26 | **(p value)** |
| Average FD-RMS motion estimation, mean SD) | 0.10 (0.05) | 0.08 (0.04) | *t* 1.44 (0.15) |
| Average DVARS-RMS estimation, mean SD) | 5.97 (0.84) | 5.84 (0.91) | *t* 0.23 (0.81) |
| Number of frame outliers, metric FD-MRS | 19.9 (10.12) | 19.52 (9.5) | *t* 0.14 (0.88) |
| Number of frame outliers, metric DVARS | 14.4 (6.96) | 14.4 (9.14) | *t* 0.02 (0.98) |
| Rotation X | 0.11 (1.78) | 0.45 (1.61) | *t* -0.73 (0.46) |
| Rotation Y | -0.26 (0.60) | 0.13 (0.54) | *t* -1.03 (0.30) |
| Rotation Z | 0.25 (1.53) | -0.57 (1.69) | *t* 1.87 (0.06) |
| Translation X | -0.03 (0.19) | 0.02 (0.12) | *t* -1.29 (0.20) |
| Translation Y | -0.10 (0.48) | 0.003 (0.13) | *t* -1.09 (0.27) |
| Translation Z | -0.007 (0.22) | 0.009 (0.13) | *t* -0.31 (0.75) |

FD, frame displacement; RMS, root mean square; DVARS, D is temporal derivative of time courses and VARS is RMS variance over voxels.

# **Table S5. Temporal irreversibility values in extremely preterm (EPT) and term-born groups (all data included)**

| **Network** | **EPT (mean ± SD)** | | **Term**  **mean ± SD** | | **p-value** | | **FDR-corrected p-value** | **Cohen’s d** | |
| --- | --- | --- | --- | --- | --- | --- | --- | --- | --- |
| Global | 1.321 ± 0.427 | | 1.588 ± 0.628 | | 0.0036 | | **0.0096** | **0.49** | |
| Default mode | 2.169 ± 1.489 | | 2.615 ± 1.404 | | 0.0022 | | **0.0088** | **0.31** | |
| Salience | 2.540 ± 1.539 | | 2.881 ± 1.387 | | 0.0099 | | **0.0158** | **0.23** | |
| Dorsal attention | 1.481 ± 0.719 | | 1.919 ± 0.945 | | 0.0095 | | **0.0158** | **0.54** | |
| Control | 1.681 ± 0.968 | | 2.454 ± 1.738 | | 0.0013 | | **0.0088** | **0.55** | |
| Visual | 4.156 ± 3.124 | | 4.486 ± 3.238 | | 0.2071 | | 0.2367 | 0.10 | |
| Somatomotor | 4.102 ± 2.376 | | 4.153 ± 2.424 | | 0.1122 | | 0.1496 | 0.02 | |
| Limbic | 4.194 ± 4.823 | | 2.687 ± 2.054 | | 0.2420 | | 0.2420 | −0.39 | |
|  |  |  | |  | |  | |  | |

Mean irreversibility values (×10⁻³) for each large-scale network in children born extremely preterm (EPT) and term-born controls. Values correspond to mean ± SD computed using all available data. Group comparisons were performed using non-parametric permutation tests. Effect sizes (Cohen’s d) were calculated to quantify the magnitude of group differences. Both original (uncorrected) and false-discovery-rate (FDR) corrected p-values are reported; FDR-significant results are shown in bold. Results were robust to the exclusion of outliers (>1.5×IQR).

**Table S6.** **Temporal irreversibility values in extremely preterm (EPT) and term-born groups (excluding outliers)**

| **Network** | **EPT**  **mean ± SD** | | **Term**  **mean ± SD** | | **Original p-value** | | **FDR-corrected p-value** | **Cohen’s d** | |
| --- | --- | --- | --- | --- | --- | --- | --- | --- | --- |
| Global | 1.212 ± 0.246 | | 1.588 ± 0.628 | | 0.0036 | | **0.0096** | **0.80** | |
| Default mode | 1.751 ± 0.512 | | 2.615 ± 1.404 | | 0.0022 | | **0.0088** | **0.72** | |
| Salience | 2.410 ± 1.366 | | 2.881 ± 1.387 | | 0.0099 | | **0.0158** | **0.34** | |
| Dorsal attention | 1.406 ± 0.588 | | 1.919 ± 0.945 | | 0.0095 | | **0.0158** | **0.60** | |
| Control | 1.379 ± 0.442 | | 2.096 ± 1.230 | | 0.0013 | | **0.0088** | **0.74** | |
| Visual | 3.205 ± 1.299 | | 3.770 ± 2.096 | | 0.2071 | | 0.2367 | 0.29 | |
| Somatomotor | 3.340 ± 1.172 | | 3.547 ± 1.822 | | 0.1122 | | 0.1496 | 0.14 | |
| Limbic | 2.336 ± 1.442 | | 2.096 ± 1.596 | | 0.2420 | | 0.2420 | −0.17 | |
|  |  |  | |  | |  | |  | |

Mean irreversibility values (×10⁻³) for each large-scale network in children born extremely preterm (EPT) and term-born controls. Values correspond to mean ± SD after exclusion of outliers, defined as values exceeding 1.5× the interquartile range (IQR) and computed within each group and network. Group comparisons were performed using non-parametric permutation tests. Effect sizes (Cohen’s d) were calculated to quantify the magnitude of group differences. Both original (uncorrected) and false-discovery-rate (FDR) corrected p-values are reported; FDR-significant results are shown in bold.

**Table S7. Number of outliers on each group**

|  | **Network** | **EPT**  **(N outliers)** | **Term**  **(N outliers)** |  |
| --- | --- | --- | --- | --- |
|  | Global | 3 | 0 |  |
|  | Visual | 4 | 2 |  |
|  | Somatomotor | 4 | 3 |  |
|  | Dorsal attention | 1 | 0 |  |
|  | Salience / Ventral attention | 1 | 0 |  |
|  | Limbic | 5 | 1 |  |
|  | Control (Executive) | 4 | 2 |  |
|  | Default mode (DMN) | 4 | 0 |  |
|  |  |  |  |  |

Outliers (>1.5×IQR) were more frequently observed in the extremely preterm (EPT) group across several networks, reflecting greater inter-individual variability in temporal irreversibility. Importantly, exclusion of these values did not alter the direction or statistical significance of group differences, indicating that the reported effects are not driven by extreme observations.

**Table S8. Effect sizes and 95% confidence intervals for group differences in temporal irreversibility.**

| **Network** | **Cohen’s d** | **95% CI**  **(lower)** | **95% CI**  **(upper)** |
| --- | --- | --- | --- |
| Global | 0.49 | −0.03 | 1.01 |
| Default mode | 0.31 | −0.21 | 0.83 |
| Salience | 0.23 | −0.29 | 0.75 |
| Dorsal attention | 0.54 | **0.02** | 1.06 |
| Control | 0.55 | **0.03** | 1.07 |
| Visual | 0.10 | −0.41 | 0.61 |
| Somatomotor | 0.02 | −0.49 | 0.53 |
| Limbic | −0.39 | −0.91 | 0.13 |

Cohen’s d and corresponding 95% confidence intervals for group differences in temporal irreversibility. Confidence intervals are provided to quantify the precision of the estimated effect sizes and should be interpreted independently from the non-parametric permutation tests used for statistical inference. Networks showing FDR-significant group differences are highlighted in bold.

**Table S9. Node-level differences in temporal irreversibility between children born extremely preterm and term-born controls**

|  |  |  |  |  | **Mean** |  |  |  |
| --- | --- | --- | --- | --- | --- | --- | --- | --- |
| **ROI** | **Network**  **Index** | **ROI_label** | **Mean Controls** | **Mean**  **EPT** | **Difference**  **Control-EPT** | **Permutation**  ***P* value** | **FDR-corrected**  ***P* value** | **Cohen’s d** |
|  |  |  |  |  |  |  |  |  |
|  |  |  |  |  |  |  |  |  |
| 1 | 1 | 7Networks_LH_Vis_1 | 0,002843151 | 0,001783968 | 0,001059183 | 0,014985015 | 0,146007838 | -0,590904951 |
| 19 | 3 | 7Networks_LH_DorsAttn_Post_4 | 0,002726706 | 0,002006248 | 0,000720457 | 0,04995005 | 0,203796204 | -0,426384915 |
| 22 | 3 | 7Networks_LH_DorsAttn_PrCv_1 | 0,003368642 | 0,002278217 | 0,001090425 | 0,03996004 | 0,181636545 | -0,49907494 |
| 28 | 4 | 7Networks_LH_SalVentAttn_Med_1 | 0,004500018 | 0,002754603 | 0,001745415 | 0,024975025 | 0,146911912 | -0,556263453 |
| 30 | 4 | 7Networks_LH_SalVentAttn_Med_3 | 0,00364622 | 0,002355229 | 0,001290991 | 0,028971029 | 0,1524791 | -0,492078152 |
| **36** | **6** | **7Networks_LH_Cont_pCun_1** | **0,003329838** | **0,001888345** | **0,001441493** | **0,000999001** | **0,04995005** | **-0,924458755** |
| 38 | 7 | 7Networks_LH_Default_Temp_1 | 0,004034813 | 0,002411247 | 0,001623566 | 0,008991009 | 0,146007838 | -0,65540216 |
| 41 | 7 | 7Networks_LH_Default_Par_2 | 0,0043747 | 0,003003126 | 0,001371574 | 0,028971029 | 0,1524791 | -0,507670333 |
| 51 | 1 | 7Networks_RH_Vis_1 | 0,002715496 | 0,001984417 | 0,000731079 | 0,044955045 | 0,195456717 | -0,444623424 |
| 69 | 3 | 7Networks_RH_DorsAttn_Post_3 | 0,002453171 | 0,001759933 | 0,000693238 | 0,016983017 | 0,146007838 | -0,552773823 |
| 71 | 3 | 7Networks_RH_DorsAttn_Post_5 | 0,003150068 | 0,002020443 | 0,001129625 | 0,022977023 | 0,146911912 | -0,546958689 |
| 72 | 3 | 7Networks_RH_DorsAttn_PrCv_1 | 0,003766767 | 0,002437079 | 0,001329688 | 0,013986014 | 0,146007838 | -0,614200989 |
| 77 | 4 | 7Networks_RH_SalVentAttn_Med_1 | 0,002852079 | 0,002057432 | 0,000794647 | 0,098901099 | 0,267817289 | -0,333769454 |
| 78 | 4 | 7Networks_RH_SalVentAttn_Med_2 | 0,005407679 | 0,003761609 | 0,00164607 | 0,024975025 | 0,146911912 | -0,556215952 |
| 83 | 6 | 7Networks_RH_Cont_PFCl_1 | 0,002669765 | 0,001587633 | 0,001082132 | 0,001998002 | 0,066600067 | -0,737610618 |
| 84 | 6 | 7Networks_RH_Cont_PFCl_2 | 0,003224519 | 0,002172634 | 0,001051886 | 0,023976024 | 0,146911912 | -0,512220792 |
| 85 | 6 | 7Networks_RH_Cont_PFCl_3 | 0,003282501 | 0,002201502 | 0,001080999 | 0,018981019 | 0,146007838 | -0,578122668 |
| 86 | 6 | 7Networks_RH_Cont_PFCl_4 | 0,003450362 | 0,001858113 | 0,001592249 | 0,015984016 | 0,146007838 | -0,601612469 |
| 89 | 6 | 7Networks_RH_Cont_pCun_1 | 0,003496427 | 0,002166079 | 0,001330348 | 0,002997003 | 0,074925075 | -0,805913456 |
| 92 | 7 | 7Networks_RH_Default_Temp_2 | 0,002656321 | 0,001625506 | 0,001030815 | 0,011988012 | 0,146007838 | -0,566838512 |
| 94 | 7 | 7Networks_RH_Default_PFCv_1 | 0,00384944 | 0,002256661 | 0,001592779 | 0,012987013 | 0,146007838 | -0,582370872 |
| 95 | 7 | 7Networks_RH_Default_PFCv_2 | 0,002937195 | 0,001875252 | 0,001061942 | 0,033966034 | 0,16983017 | -0,50478506 |
| 96 | 7 | 7Networks_RH_Default_PFCdPFCm_1 | 0,003968318 | 0,002925901 | 0,001042417 | 0,035964036 | 0,171257314 | -0,489312067 |
| **97** | **7** | **7Networks_RH_Default_PFCdPFCm_2** | **0,00460801** | **0,002662655** | **0,001945355** | **0,000999001** | **0,04995005** | **-0,951633674** |
|  |  |  |  |  |  |  |  |  |
|  |  |  |  |  |  |  |  |  |

The table includes all cortical parcels reaching the uncorrected permutation threshold. Positive mean differences (Controls − EPT) indicate lower temporal irreversibility in children born extremely preterm. No parcels showing higher temporal irreversibility in the EPT group reached the uncorrected significance threshold. Nodes remaining significant after false discovery rate correction across the 100 cortical parcels are shown in **bold**. **ROI**, region of interest; **EPT**, extremely preterm; **FDR**, false discovery rate. Network abbreviations follow the Schaefer 2018, 7-network parcellation: **Vis**, visual network; **SomMot**, somatomotor network; **DorsAttn**, dorsal attention network; **SalVentAttn**, salience/ventral attention network; **Limbic**, limbic network; **Cont**, control (frontoparietal) network; **Default**, default mode network. Hemisphere abbreviations: **LH**, left hemisphere; **RH**, right hemisphere.
